# Diagnosis of Type IV Endoleak After Endovascular Aneurysm Repair Using Visualization With Novel Software

**DOI:** 10.7759/cureus.60527

**Published:** 2024-05-17

**Authors:** Hiroyuki Nishi, Mutsunori Kitahara, Takura Taguchi, Masao Yoshitatsu

**Affiliations:** 1 Department of Cardiovascular Surgery, National Hospital Organization, Osaka National Hospital, Osaka, JPN

**Keywords:** novel visual software, endoleak, computed tomography, type iv endoleak, endovascular aneurysm repair

## Abstract

A Type IV endoleak is a very rare complication following endovascular aneurysm repair (EVAR) and differential diagnosis can be difficult. Reported here is a case that showed the development of a Type IV endoleak after an EVAR procedure, for which a novel software was useful to differentiate that from Type I based on visual confirmation. The 89-year-old man was diagnosed with a large abdominal aortic aneurysm, sized 70 mm, as shown by computed tomography (CT). EVAR was performed in a routine fashion using an Endurant II stent graft. Postoperative CT revealed a massive endoleak around the neck that was difficult to differentiate between Types I and IV. The use of the novel software Viewtify (SCIEMENT, Inc., Tokyo, Japan) to visualize the endoleak with surrounding tissues as real-time three-dimensional computer graphics (3DCG) resulted in confirmation that the endoleak was not from the proximal end but rather the stent graft body. CT findings obtained one week later showed that the endoleak had diminished and no additional procedures were needed. Following a diagnosis of endoleak after EVAR, images viewed with Viewtify helped to confirm the appropriate diagnosis. This novel software was found useful to clarify the position and mechanism of a Type IV endoleak.

## Introduction

A Type IV endoleak is a very rare complication following endovascular aneurysm repair (EVAR) [1] and diagnosis can be difficult. Should a definitive diagnosis of such an endoleak be made, appropriate follow-up procedures can be performed, because a Type IV endoleak sometimes spontaneously disappears at one to two months after EVAR [2,3]. However, for cases with a Type I or III endoleak, prompt treatment is required, thus determination of endoleak type is very important.

Viewtify® (SCIEMENT, Inc., Tokyo, Japan) is a newly developed software that can synthesize three-dimensional computer graphics (3DCG) from computed tomography (CT) images, make cross-sections, and provide the 3DCG as stereoscopic images by using a naked-eye, eye-sensing stereoscopic display ELF-SR1 or ELF-SR2 (SONY, Tokyo, Japan), all in real-time. Additionally, the software can change the threshold CT value for 3DCG reconstruction manually in real-time, which enables the real-time adjustment of 3DCG visualization and was used to visualize complex congenital heart disease [4]. Viewtify offers only basic medical image processing, and thresholding by CT value, but excels in speed, generating 3DCG in just 0.01 to 0.03 seconds after specifying the threshold, making it 10 to 100 times faster than other software. It enables real-time fine-tuning of thresholds. Additionally, Viewtify employs surface rendering instead of volume rendering for enhanced depth perception and spatial relationships.

In the present case, a definitive diagnosis of Type IV was attained based on image evaluation findings obtained with Viewtify for a postoperative EVAR case in which it was difficult to distinguish between Types I and IV.

## Case presentation

**Figure 1:**
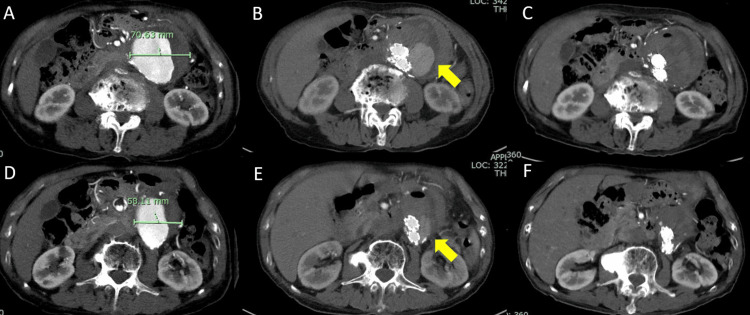
Contrast-enhanced computed tomography findings (A, D) Preoperative imaging. (B, E) Images obtained on day 5 after surgery. (C, F) Images obtained on day 12 after surgery. Preoperative contrast-enhanced computed tomography revealed large abdominal aortic aneurysm (A: same level of B and C, D: same level of E and F). Five days after surgery, a large amount of contrast medium leakage was observed in the aneurysm (arrows, B, E). The contrast medium in the aneurysm had disappeared 12 days after the operation (C, F).

An 89-year-old male, for whom an abdominal aortic aneurysm had been previously pointed out without follow-up examinations, underwent a CT examination, which showed the aneurysm to be quite large. The patient had a history of hypertension, diabetes, cerebral infarction, and COPD requiring home oxygen. A procedure was scheduled, and preoperative CT showed the aneurysm to be 70 mm in diameter with a saccular shape, a landing zone under the renal arteries, and a diameter of 12 mm in the left and right common iliac arteries (Figures 1A-1F). The anatomy was found to be favorable for EVAR, which was performed with an Endurant II (Medtronic Inc., Minnesota, USA) under local anesthesia.

**Figure 2:**
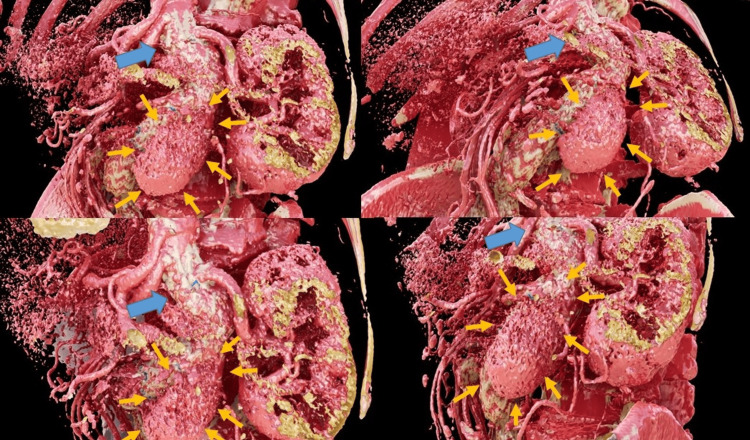
Postoperative reconstructed 3DCG images using Viewtify from the CT images of day 5 after surgery The endoleak was visualized stereoscopically (orange arrow). It was found to be far from the central end of the stent graft (thick blue arrow) and leaking from the body of the stent. The image is shown as a non-stereoscopic image, while stereoscopic 3D view provided clear observation of the conditions.

The early course following the procedure was uneventful. However, postoperative CT on postoperative day (POD) 5 showed leakage of contrast medium into the aneurysm and a relatively massive endoleak was confirmed (Figures 1A-1F). The endoleak appeared to be connected to the neck of the aneurysm, though it was difficult to diagnose the type due to an angulated aneurysm neck and a huge aneurysm sac. Since the aneurysm was large and the endoleak massive, it was important to distinguish between Type I and Type IV endoleak, thus an image was constructed using Viewtify with the CT images. Thin-slice CT images are processed rapidly on the GPU to construct high-quality, stereoscopic 3DCG images displayed on a naked-eye stereoscopic display. The results clarified blood gushing out from the vicinity of the leg of the stent graft into the aneurysm and that it was separated from the neck (Figure 2, Video 1). Therefore, a Type IV endoleak was determined. Follow-up examinations were continued without further treatment and the endoleak was not seen in CT findings obtained one week later. The patient was discharged without incident and no endoleak was observed in CT imaging during the long-term postoperative period.

**Video 1:** Video of Viewtify This video file demonstrates the relationship between the endoleak and stent graft in the present case in a more three-dimensional and detailed manner.

## Discussion

A Type IV endoleak is defined as early leakage through the pores of an otherwise intact endovascular graft [5], though it is very rare, with a reported frequency of approximately 0.3%. In general, a reoperation is not generally required even long after the performance of an EVAR, thus follow-up examinations alone are often chosen [6]. On the other hand, when an endoleak is observed following that repair procedure for a large aneurysm, differential diagnosis is very important and additional treatment may be required in some cases. In particular, Type I and Type III endoleaks are highly likely to lead to serious complications [7], thus prompt diagnosis is necessary. In the present patient, an endoleak was observed after EVAR surgery for a huge aneurysm sized 70 mm and it was necessary to identify the cause as soon as possible. CT findings showed that the leakage site was close to the neck site, making it difficult to completely deny Type I because the neck was angulated and the endoleak was massive. A Type IV endoleak is usually observed without apparent contrast extravasation [8,9]. In the present case, the use of Viewtify and the stereoscopic display resulted in very clear 3DCG visualization. It thus became clear that the leak was from the stent graft and Type I could be ruled out, thus the option of follow-up examinations alone was possible. The reconstructed 3DCG of the present case clearly depicted a Type IV endoleak.

In general, contrast-enhanced CT is useful when an endoleak has occurred, though it is often very difficult to differentiate between types for unequivocal determination. In such cases, contrast-enhanced echocardiography is extremely useful, and a previous report noted that it is possible to visualize endoleak sites [10]. Furthermore, another study noted that late-phase magnetic resonance imaging with a blood pool agent to visualize graft porosity was useful in detecting a Type IV endoleak [11]. However, using a combination of various modalities is complicated and the superiority of echocardiography for such detection has not been demonstrated [12]. Findings concerning accurate detection of Type IV endoleaks are lacking, thus the present results showing the ability to make a definitive diagnosis using this novel software with contrast-enhanced CT imaging are considered to be clinically useful.

Viewtify performs nearly all of its calculations on the graphics processing unit (GPU) of the computer, thus enabling real-time 3DCG synthesis from CT images. It was developed using Unreal Engine (Epic Games, North Carolina, USA), a game engine, that makes high-quality 3DCG rendering possible in real-time. A report from Omori et al. noted the use of Viewtify to visualize the intracardiac structure of a complex double outlet right ventricle by adjusting 3DCG in real-time for evaluations of the position of the ventricular septal defect and subaortic stenosis, which confirmed that biventricular repair was possible. In the present case, 3D images were obtained with Viewtify, which made it possible to visualize the appearance of the endoleak while viewing it from any angle on the stereoscopic display, leading to a diagnosis of Type IV. Finding the optimal CT threshold value for visualizing endoleak as 3DCG can be a trial-and-error process. Using Viewtify, which allows real-time threshold adjustments, may enhance the visualization of endoleak more effectively. We believe that this new software has potential usefulness for identifying various types of endoleaks, Type IV in particular, and clarifying the related mechanism through visualization of occurrence site and morphology.

Limitations include the fact that thin slice data from CT is required, the image quality is entirely dependent on the information obtained from CT, and there may be the effect of halation from metals. Further research is needed to analyze and differentiate images of different types of endoleaks using a large number of cases.

## Conclusions

For diagnosis of endoleak occurring after an EVAR procedure, imaging obtained with the use of the novel software Viewtify was very helpful in confirming an appropriate diagnosis. 3D images made it possible to visualize the appearance of the endoleak from any angle. The present results showed the ability to make a definitive diagnosis using this novel software. It is considered to have great usefulness for clarification of the position and mechanism of a Type IV endoleak. More detailed analysis by endoleak type using a large number of cases is required.
